# Current experimental disease-modifying therapeutics for multiple system atrophy

**DOI:** 10.1007/s00702-021-02406-z

**Published:** 2021-08-16

**Authors:** Miguel Lemos, Gregor K. Wenning, Nadia Stefanova

**Affiliations:** Laboratory for Translational Neurodegeneration Research, Division of Neurobiology, Department of Neurology, Medical University of Innsbruck, Innsbruck, Austria

**Keywords:** Multiple system atrophy, Synuclein, Neuroinflammation, Therapy, Small molecules, Immunotherapy

## Abstract

Multiple system atrophy (MSA) is a challenging neurodegenerative disorder with a difficult and often inaccurate early diagnosis, still lacking effective treatment. It is characterized by a highly variable clinical presentation with parkinsonism, cerebellar ataxia, autonomic dysfunction, and pyramidal signs, with a rapid progression and an aggressive clinical course. The definite MSA diagnosis is only possible post-mortem, when the presence of distinctive oligodendroglial cytoplasmic inclusions (GCIs), mainly composed of misfolded and aggregated α-Synuclein (α-Syn) is demonstrated. The process of α-Syn accumulation and aggregation within oligodendrocytes is accepted one of the main pathological events underlying MSA. However, MSA is considered a multifactorial disorder with multiple pathogenic events acting together including neuroinflammation, oxidative stress, and disrupted neurotrophic support, among others. The discussed here treatment approaches are based on our current understanding of the pathogenesis of MSA and the results of preclinical and clinical therapeutic studies conducted over the last 2 decades. We summarize leading disease-modifying approaches for MSA including targeting α-Syn pathology, modulation of neuroinflammation, and enhancement of neuroprotection. In conclusion, we outline some challenges related to the need to overcome the gap in translation between preclinical and clinical studies towards a successful disease modification in MSA.

## Multiple system atrophy: from pathology to clinical presentation

The term multiple system atrophy (MSA) was introduced in 1969 by Graham and Oppenheimer, who recognized the overlapping pathology of three disorders, namely striatonigral degeneration (SND), olivopontocerebellar atrophy (OPCA) and Shy–Drager syndrome (Graham and Oppenheimer 1969). Later, in 1989, Papp and colleagues strengthened the definition of MSA with the discovery of glial cytoplasmic inclusions (GCIs) in the brains of these patients. Finally, in 1998, it was observed that the MSA-specific GCIs were mainly composed of misfolded and aggregated α-Synuclein (α-Syn) (Gai et al. 1998; Spillantini et al. 1998; Wakabayashi et al. 1998).

Over the years, environmental and genetic factors have been studied to understand the disease etiology (Sturm and Stefanova 2014; Jellinger and Wenning 2016). *COQ2* gene polymorphisms and genetic variants have been associated with MSA in East Asian population (Fujioka et al. 2014; Ogaki et al. 2014; Chen et al. 2015). However, the same variants were not confirmed in MSA patients from Europe or North America (Sailer et al. 2016). The presence of mutations, duplications and triplications of the *SNCA* gene*,* encoding α-Syn, in familial cases showing features of MSA or Parkinson’s disease (PD) arose the question whether *SNCA* was associated with MSA. Polymorphisms of the *SNCA* locus were identified in some European MSA patients; however, such observations were not replicated in larger cohorts of pathologically confirmed MSA cases (Al-Chalabi et al. 2009; Scholz et al. 2009). A genome-wide association study (GWAS) reported in 2016 no association of *COQ2* and *SNCA* with MSA, but several potential interesting candidates were identified, highlighting the need for further genome studies with larger and well-characterized MSA samples to understand the genetics of this disorder (Sailer et al. 2016). Recently, a GWAS summary statistics study of MSA and seven autoimmune diseases identified a shared genetic etiology between MSA and inflammatory bowel disease (Shadrin et al. 2020). These findings reinforced the role of neuroinflammation and the gut-brain axis in association with a possible polygenic predisposition in the pathophysiology MSA.

Neuropathologically, MSA is defined by characteristic lesions in the striatonigral and olivopontocerebellar systems, which correlate with the two major motor phenotypes of MSA with predominant parkinsonism (MSA-P) and predominant cerebellar ataxia (MSA-C), respectively (Fanciulli and Wenning 2015). In detail, the main affected brain regions in MSA-P include the caudate nucleus and the dorsolateral putamen as well as the substantia nigra, the globus pallidus and the subthalamic nucleus (Wenning and Quinn 1997; Ozawa et al. 2004; Jellinger 2014). Patients with MSA-P show motor features of parkinsonism including slowness of movements, rigidity, and a tendency to fall. The degeneration of the striatal regions is associated with a poor response to levodopa treatment, which sometimes helps clinicians as a diagnostic criterion and a distinction to PD (Fanciulli and Wenning 2015). In MSA-C, severe degeneration of cerebellar Purkinje neurons, as well as neurons in the pontine nuclei and inferior olivary nucleus results in cerebellar features including wide-based gait, uncoordinated limb movements and action tremor (Wenning et al. 1996; Jellinger et al. 2005; Fanciulli and Wenning 2015). MSA is also characterized by early and progressive autonomic failure, associated with neurodegenerative changes affecting preganglionic autonomic centers in the brain stem and the spinal cord (Ozawa 2007; Ahmed et al. 2012). The most common non-motor symptoms include the urogenital and cardiovascular features, including urinary and sexual dysfunction and severe neurogenic orthostatic hypotension, respectively. Respiratory disturbances and sleep disorders, namely REM sleep behavior disorder and central sleep apnea that can lead to the sudden death are also observed in MSA patients (Beck et al. 1994; Benarroch et al. 2006; Tada et al. 2007; Palma et al. 2015; McKay and Cheshire 2018; Ueda et al. 2020). Existing symptomatic therapies provide only limited amelioration of some functional deficits, e.g., Parkinsonism, cerebellar ataxia, and autonomic failure (Coon and Ahlskog 2021). The modest benefit and poor validation of the majority of the treatments in MSA is one of the drivers for the search of new avenues for disease modification in this complex disorder.

The main pathological hallmark of MSA is the presence of proteinaceous oligodendroglial cytoplasmic inclusions (GCIs, initially named Papp–Lantos bodies). In addition, it is possible to observe the presence of oligodendroglial nuclear inclusions as well as neuronal axonal, cytoplasmic and nuclear inclusions (Papp et al. 1989; Yoshida 2007). Such neuronal inclusions were identified to be widespread in brain regions not previously implicated in the disease pathology, thus suggesting a role in the development and progression of MSA (Cykowski et al. 2015). Nevertheless, GCIs represent the most abundant type of inclusions in the brain of MSA patients, and their diffuse presence is an absolute requirement for the definite post-mortem diagnosis of MSA (Trojanowski and Revesz 2007). Besides oligodendroglial dysfunction and selective neurodegeneration, prominent neuroinflammation and demyelination are found in the brains of MSA patients (Jellinger 2014; Brettschneider et al. 2017). Post-mortem analysis reveals the presence of activated microglia and reactive astrocytes associated with α-Syn inclusion pathology and neurodegeneration (Ozawa et al. 2004; Ishizawa et al. 2008).

## MSA pathogenesis: identifying putative targets for disease modification

The pathogenic mechanisms underlying MSA remain inconclusive. Nevertheless, converging evidence collected from post-mortem studies and preclinical models classifies MSA as a primary oligodendrogliopathy, included in the category of α-Synucleinopathies together with PD and dementia with Lewy Bodies (DLB) (Spillantini and Goedert 2000). The presence of α-Syn aggregates in the cytoplasm of oligodendrocytes defines MSA as a unique α-Synucleinopathy, since in PD and DLB, α-Syn pathology occurs mostly in the cytoplasm of neurons (Trojanowski and Revesz 2007; Goedert et al. 2017). These observations result in several questions that we cannot completely answer so far. For instance, what is the source of α-Syn within oligodendrocytes? Are the α-Syn inclusions a disease trigger or an epiphenomenon? Where should we focus when defining therapeutic targets in MSA?

### Targeting α-Synuclein pathology

α-Syn is an intrinsically disordered protein widely distributed in the mammalian central nervous system, more precisely in the pre-synaptic terminals of neurons, with a suggested physiological role in synaptic homeostasis, vesicle recycling and synaptic neurotransmission (Maroteaux et al. 1988; Jakes et al. 1994; Iwai et al. 1995). Under pathological conditions, such as α-Syn mutations, multiplications, and post-translational modifications or stress-induced changes in the cellular environment, α-Syn becomes highly prone to aggregate into pathological oligomeric and large fibrillary structures such as GCIs and Lewy Bodies (LBs) (Spillantini et al. 1998; Anderson et al. 2006; Auluck et al. 2010; Wales et al. 2013). To date, the process of α-Syn accumulation and aggregation is considered one of the main pathological events underlying α-Synucleinopathies, leading to neuronal dysfunction, neuroinflammation and neurodegeneration (Mahul-Mellier et al. 2020). Specifically, pathological forms of α-Syn seem to interact with cellular components and pathways affecting the cellular homeostasis. For example, pathological α-Syn inhibits lysosomal and proteasome activity, impairs axonal transport, induces calcium dyshomeostasis and increases the production of reactive oxygen species associated with the damage of the mitochondria (Stefanis et al. 2001; Danzer et al. 2007; Volpicelli-Daley et al. 2011; Mazzulli et al. 2016; Burré et al. 2018; Ganjam et al. 2019). In addition, the aggregation into larger fibrillary species has been shown to impair important protein quality control mechanisms that might aggravate the neurodegenerative process (Djajadikerta et al. 2020).

Recently, it was observed that in the process of α-Syn aggregation, the formation of different fibrillary strains can arise in MSA as compared to PD or DLB (Schweighauser et al. 2020; Shahnawaz et al. 2020). It is believed that the cellular environment might play an important role in the formation of the different strains with disease-specific biochemical and proteolytic profiles (Peng et al. 2018) and a more rapid transmissibility in a predisposing molecular and cellular background (Watts et al. 2013). Novel data suggest the role of p25α, a myelin protein, for the formation of a more aggressive prodegenerative α-Syn strain with possible relevance to MSA (Ferreira et al. 2021). Intriguingly, the structures of in vitro seeded assemblies of recombinant α-Syn differ from those of the MSA-derived seeds further reinforcing the role of the specific cellular environment for the formation of disease-specific strains (Lövestam et al. 2021).

In summary, the prevention of α-Syn aggregation as well as the enhancement of its degradation constitute promising therapeutic strategies for disease modification in MSA. The disease-specific strains identified in MSA vs PD and DLB further suggest the possible need for individualized approaches of the therapies targeting α-Syn.

Since it became widely accepted that α-Syn accumulation, spreading and aggregation constitute major driving forces leading to neurodegeneration, a majority of the developed therapeutic approaches has been focused on enhancing α-Syn degradation and prevention or disruption of its aggregation (Brundin et al. 2017). To enhance the degradation of α-Syn, initial studies were based on inducing α-Syn degradation by stimulating macroautophagy using rapamycin, lithium and nilotinib. Lithium was one of the earliest candidates in a clinical trial with MSA patients; however, it had to be interrupted due to severe adverse effects (Saccà et al. 2013). Nilotinib showed neuroprotective effects in a mouse model of PD, but failed in the PLP-α-Syn MSA mouse model, a transgenic mouse overexpressing α-Syn in oligodendrocytes under the proteolipid protein promoter (PLP) (Hebron et al. 2013; Lopez-Cuina et al. 2020). A phase II, double-blind clinical trial with Sirolimus (rapamycin) in MSA patients has been completed (NCT03589976), but no results are published yet.

Another approach to increase the extracellular a-Syn clearance is to promote its degradation by microglial cells. Experimental studies showed that Toll-like receptor 4 (TLR4), a member of highly conserved molecules that recognize pathogen-associated molecular patterns, played an important role in the microglial α-Syn clearance suggesting that up-regulation of TLR4 in microglia may constitute a relevant target for MSA therapy (Stefanova et al. 2011; Fellner et al. 2013). Thereafter, Venezia and colleagues used Monophosphoryl lipid A (MPLA), a TLR4 selective agonist and vaccine component with lower pro-inflammatory toxicity to test the hypothesis in PLP-α-Syn mice. Results showed that the administration of MPLA led to a significant motor improvement, preservation of nigral dopaminergic neurons and decreased levels of GCIs (Venezia et al. 2017).

**Fig. 1:**
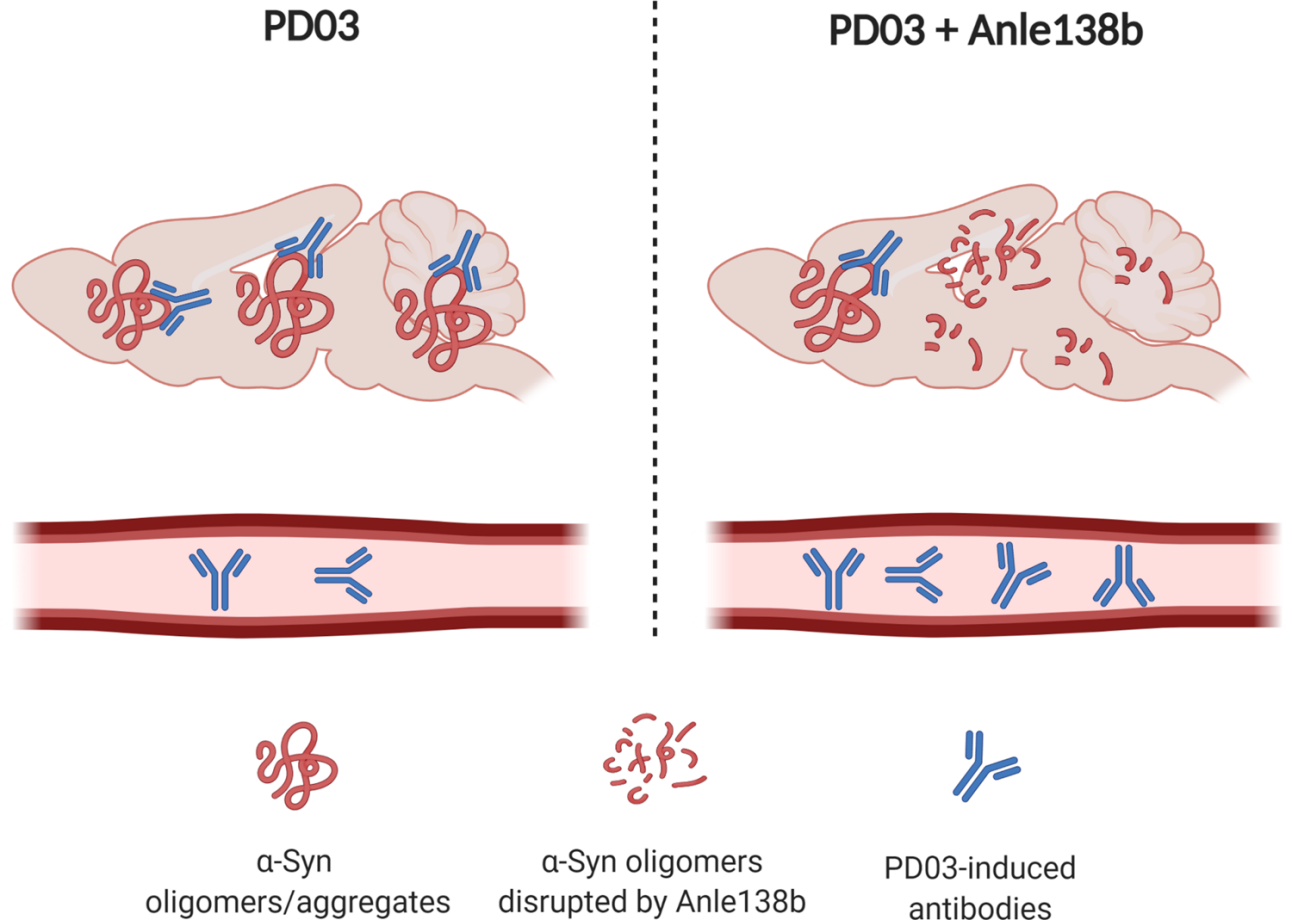
Hypothesis: compartmental redistribution of anti-α-Syn antibodies after specific immunotherapy. PD03 immunization of PLP-α-Syn mice results in significant binding of the specific antibodies in the brain. This antibody binding strongly decreases, while higher levels of free antibodies in the plasma are detected when the specific α-Syn oligomers,—the target of the PD03 antibodies—, are reduced by Anle 138b (Lemos et al 2020). The shift between specific antibody binding and free antibodies in the plasma may be relevant to the definition of immunogenicity in clinical trials. Created with BioRender.com

The use of immunotherapy for the treatment of neurodegenerative disorders has attracted the attention of researchers and pharmaceutical companies towards the development of anti-α-Syn immunotherapies to enhance its degradation and clearance. The principle of immunotherapy (passive or active) is based on the specific binding of the antigen α-Syn and its respective antibody, followed by clearance of the complexes (Mandler et al. 2015; El-Agnaf et al. 2017). Active immunization for targeting the α-Syn pathology has been carried out with short synthetic peptide fragments (AFFITOPEs®), mimicking parts of the native sequence and structure of the human α-Syn protein. The immunogenic peptide, i.e., AFFITOPE, operates a B cell epitope and is responsible for the specificity of the immune response. Initial studies in PD, DLB, and MSA mouse models demonstrated the efficacy of the AFFITOPE® PD01 and PD03, which triggered specific antibody generation with CNS penetration and lowered α-Syn aggregates and oligomers, leading to neuroprotection and improvement of locomotor behavior in PD and MSA mice, respectively (Mandler et al. 2014, 2015; Lemos et al. 2020). The first phase I clinical trial with PD patients using PD01 suggested good immunogenicity, safety and tolerability (Volc et al. 2020). In 2020, a clinical trial with PD01 and PD03 in MSA patients showed that both AFFITOPEs® presented immunogenicity and good tolerability. However, the authors noticed that the antibody levels in the plasma were higher in individuals receiving PD01 than in patients receiving PD03 (Meissner et al. 2020). In contrast, substantial levels of PD03-induced antibodies were reported in PD patients (Poewe et al. 2021). These differences in plasma antibody levels between PD and MSA patients receiving PD03 immunotherapy and the evidence of different α-Syn strains in PD and MSA (Schweighauser et al. 2020; Shahnawaz et al. 2020), led the researchers to speculate that vaccines may have different binding of the antibodies to disease-specific α-Syn conformations, which may result in the observed difference in plasma antibody levels reported in the clinical trials in PD and MSA. In support of this hypothesis, PD03 immunotherapy in MSA mice showed high binding of IgGs in the brain, suggesting that the PD03-induced antibodies entered the brain and accumulated at the sites of α-Syn pathology. In contrast, if the MSA mice received in parallel to PD03 also Anle138b, a small molecule that modulates the oligomerization of α-Syn, the IgG binding in the brain was significantly decreased (Heras-Garvin et al. 2019; Lemos et al. 2020). This phenomenon was accompanied by increase in the measured plasma antibodies to α-Syn in the mice receiving combined therapy, therefore, supporting the hypothesis that the plasma levels of anti-α-Syn antibodies may reflect not simply the immunogenicity of the used vaccine, but also the level of selective binding to a specific α-Syn conformation (Fig. 1). Taken together, the clinical and preclinical evidence reinforce the relevance of disease-specific α-Syn strains for the efficacy of active immunotherapy in α-Synucleinopathies.

Approaches of passive immunization to target α-Syn pathology using antibodies targeting different α-Syn species have been extensively tested preclinically in PD models (Zella et al. 2019). Respectively, several clinical trials testing passive immunization in PD have been launched. First preclinical data supporting the efficacy of passive immunization approach in MSA were also reported and the clinical application in MSA is currently discussed (Kallab et al. 2018).

The use of small molecules such as Anle138b has shown promising results in preventing α-Syn accumulation or disrupting the formation of toxic oligomeric species. Anle138b is a small compound that can be delivered orally and crosses the blood–brain barrier. Preclinical studies with Anle138b in a MSA mouse model suggested neuroprotection associated with decreased α-Syn oligomerization, lower microglial activation and resulting in motor improvement (Heras-Garvin et al. 2019). Recently, a phase I clinical trial with Anle138b in healthy patients was successfully completed (NCT04208152) and confirmed its safety. A follow-up phase II study in MSA patients is currently in preparation.

Several other α-Syn aggregation inhibitors were developed aiming to disrupt the oligomerization process of α-Syn. The small molecule ATH434 (former PTB434) is a moderate iron chelator shown to reduce α-Syn accumulation by redistributing labile iron in the brain. The treatment with ATH434 in a mouse model of MSA showed preservation of dopaminergic neurons, lowering of ferric iron in the brain and reduced α-Syn oligomerization, resulting in improvement of the motor deficits (Heras-Garvin et al. 2021). Importantly, a phase I clinical trial of ATH434 in healthy volunteers has been completed demonstrating the safety of the compound (Stamler et al. 2019, 2020).

In the preclinical stage, two more therapeutic candidates are being evaluated for possible disease-modifying strategies in MSA, including the molecular tweezer CLR01 and the caspase-1 inhibitor VX-765. CLR01 is a small molecule that binds lysine residues of α-Syn, and therefore, disrupts already formed oligomers and prevents the formation and new high-molecular-weight assemblies (Attar et al. 2014). CLR01, when delivered intracerebroventricular in PLP-α-Syn mice, showed reduced formation of oligomeric α-Syn species accompanied by rescue of dopaminergic neurons in the substantia nigra of treated mice supporting the feasibility of the strategy (Herrera-Vaquero et al. 2019). Currently, better delivery options for molecular tweezers are explored.

Alternatively, the process of C-terminal truncation of α-Syn by caspase-1 has been shown to mediate α-Syn aggregation (Li et al. 2005; Ulusoy et al. 2010). By inhibiting caspase-1 activity with VX-765 in PLP-α-Syn mice, Bassil and colleagues observed a neuroprotective effect associated with motor improvement and reduced α-Syn monomeric and oligomeric levels (Bassil et al. 2016). VX-765 has already shown promising results in clinical applications (CT01048255 and NCT0150138) that further supports its development for clinical trials in MSA.

Two other approaches targeting α-Syn aggregation, rifampicin and Epigallocatechin gallate (EGCG), a green tea extract, have been already tested in MSA patients, failing to show disease modification (Low et al. 2014; Levin et al. 2019). Rifampicin is an antibiotic used for the therapy of tuberculosis and leprosy and suggested to inhibit the formation of α-Syn fibrils. The strategy was encouraged by positive effects of rifampicin in the MBP-α-Syn mouse (Ubhi et al. 2008), however, no efficacy was observed in a phase II clinical trial in MSA. Among the reasons for the failure of the strategy, severe adverse events as well as the advanced disease stages of treatment initiation have been discussed.

The use of antisense oligonucleotides (ASOs) is an alternative therapeutic approach to suppress the production of α-Syn, and therefore, reduce its intracellular toxic accumulation. Preclinical studies targeting the SNCA gene product have demonstrated encouraging results in PD models (Alarcón-Arís et al. 2018). A phase I clinical trial to assess safety and tolerability of BIIB101, an antisense oligonucleotide targeting *SNCA* mRNA, is currently ongoing in European MSA patients (NCT04165486).

In summary, a considerable amount of preclinical evidence supports targeting α-Syn pathology for disease modification in MSA as well as in other α-Synucleinopathies. Successful clinical trials with α-Syn targeting in MSA that prove efficacy of the approach in patients are currently awaited.

### Targeting microglia activation and neuroinflammation

Neuroinflammation is a crucial part of the neuropathological process in MSA and other α-Synucleinopathies (Zhang et al. 2005; Stefanova et al. 2007; Wilms et al. 2009; Couch et al. 2011; Refolo and Stefanova 2019). It is mediated by the activation of quiescent microglia cells that respond to neuronal damage and pathological α-Syn by secreting pro- and anti-inflammatory cytokines, chemokines and reactive oxygen species (ROS). Imaging and neuropathological studies in human brains showed that microglia activation and astrogliosis can be found at sites of α-Syn pathology in MSA (Gerhard et al. 2003; Ishizawa et al. 2004; Kiely et al. 2018; Li et al. 2018; Kübler et al. 2019). Whether such inflammatory responses are directly associated with the pathogenesis of the disease, or they represent a downstream effect triggered by the pathological accumulation of α-Syn is still under debate. In favor of the first hypothesis, a recent study in PD patients engrafted with embryonic dopaminergic neurons showed microglia activation in all grafts preceding the detection of α-Syn aggregates (Olanow et al. 2019). Furthermore, LPS treatment of PLP-α-Syn mice was shown to trigger increased formation of insoluble oligomeric α-Syn species (Venezia et al. 2017). In support, the process of chronic inflammation and microglia activation was shown to induce α-Syn aggregation and aggravate the neurodegenerative process (Lema Tomé et al. 2013). On the other hand, the pathological accumulation of α-Syn in the brain can be responsible for inducing microglia activation, cytokine release and a shift towards a pro-inflammatory intracellular environment (Fellner et al. 2011; Vieira et al. 2015; Refolo and Stefanova 2019). It was also observed that α-Syn accumulation induces the activation of reactive astrocytes, therefore enhancing the neuroinflammation process (Lee et al. 2010; Radford et al. 2015). Astrogliosis has been described at sites of α-Syn pathology in MSA brains, paralleling the neurodegenerative changes (Ozawa et al. 2004; Radford et al. 2015). α-Syn aggregates can be found in the cytoplasm of astrocytes and lead to astrocytic dysfunction (Song et al. 2009; Sorrentino et al. 2019). Altogether, these observations support the notion that α-Syn-triggered microglial activation and astrogliosis may contribute to the neurodegenerative changes in MSA, similar to other α-Synucleinopathies (Stefanova et al. 2012; Sanchez-Guajardo et al. 2015; Lim et al. 2016). Although still not well understood, the processes of microglial activation and neuroinflammation have been getting increased attention and exploited for disease-modifying therapies in MSA.

According to PET and neuropathological studies in MSA brains, the process of microglial activation and neuroinflammation can be already detected at early-disease stages (Gerhard et al. 2003; Ishizawa et al. 2004; Kübler et al. 2019; Refolo and Stefanova 2019). Such observations raise the possibility that reduction of microglial pro-inflammatory activity and anti-inflammatory strategies may represent a promising approach for disease modification in MSA. A pilot open-label study investigated the safety and preliminary efficacy of intravenous immunoglobulin as a general anti-inflammatory approach in MSA over a 6-month period. The treatment was generally well tolerated, with some signs of symptom improvement, but with no effect on brain and cerebellar atrophy in imaging analysis (Novak et al. 2012). Minocycline, a tetracycline antibiotic with brain penetrance and anti-inflammatory activity, after early application before the symptom onset in PLP-α-Syn mice, had neuroprotective effects on dopaminergic neurons in the substantia nigra associated with lower microglia activation and reduced neuroinflammation markers (Stefanova et al. 2007). However, a double-blind phase II clinical trial in clinically probable MSA patients (i.e., after motor symptom onset) replicated reduced microglia activation as suggested by the preclinical study, but failed to ameliorate the clinical progression of symptoms, probably due to the advanced disease stage of the patients (Dodel et al. 2010). Similarly, fluoxetine, a selective serotonin reuptake inhibitor with anti-inflammatory properties, although showing promising neuroprotective results in MBP-α-Syn mice, failed to change the disease progression in a double-blind phase II clinical trial in MSA patients (NCT01146548) (Ubhi et al. 2012; Valera et al. 2014). Finally, Verdiperstat is a myeloperoxidase (MPO) inhibitor, which showed neuroprotective effects in an early-disease stage MSA mouse model but failed to demonstrate the same extent of efficacy in a model of advanced disease (Stefanova et al. 2012; Kaindlstorfer et al. 2015). Currently, the M-STAR phase III clinical trial is assessing the efficacy of Verdiperstat in MSA patients (NCT03952806). Based on the preclinical data and the previous experience from clinical trials targeting neuroinflammation in MSA, one could speculated that the outcomes of the M-STAR trial will strongly depend on the disease stage of the enrolled patients.

### Targeting cellular dysfunction and loss (neuroprotection)

The selective striatonigral degeneration observed in MSA-P patients can lead to the dysfunction of corticostriatal glutamatergic, as well as striatal GABAergic projections. The impairment of such projections can result in neurotoxicity and add to the neurodegenerative cascade. On the other hand, the accumulation of α-Syn aggregates within oligodendrocytes leads to the dysfunction of these cells, hampering the neurotrophic support by brain-derived neurotrophic factor (BDNF) and glial-derived neurotropic factor (GDNF) and resulting in neuronal death (Ubhi et al. 2010). In addition, Impaired insulin/insulin-like growth factor-1 (IGF-1) signaling and insulin resistance in MSA patients, as well as increased IGF-1 brain levels in MSA mice have been reported (Ubhi et al. 2010; Numao et al. 2014; Bassil et al. 2017). Insulin and IGF-1 appear to be involved in several cellular processes including the synthesis of the myelin sheaths and oligodendrocyte maturation as well as neuronal homeostasis, thereafter their deficits lead to neurotoxicity and consequent neurodegeneration (Bassil et al. 2017). Therefore, a third line of therapeutic strategies has been directed to prevent oligodendroglial dysfunction and to provide neuroprotection of the affected neuronal populations.

Rasagiline, an irreversible inhibitor of monoamine oxidase-B that prevents the breakdown of dopamine and has anti-apoptotic effect through neuronal trophic support, appeared to induce neuroprotection and ameliorate motor deficits in very high doses in the PLP-α-Syn MSA mouse model (Stefanova et al. 2008). Due to the limitation of the applicable dose in patients, associated to its side effects, the phase II clinical trial with Rasagiline in MSA patients failed to modify the symptom progression (Poewe et al. 2015). The infusion of GDNF in an MSA mouse demonstrated an attenuation of the motor deficits, correlated with preservation of dopaminergic neurons (Ubhi et al. 2010). Currently, a phase I clinical trial with AAV2-GDNF gene therapy in MSA patients is in progress (NCT04680065). Exendin-4 (Exenatide), an anti-diabetic drug and a glucagon-like peptide-1 analogue, presented neuroprotective effects associated with decreased oligomeric α-Syn and insulin resistance in the PLP-α-Syn mouse (Bassil et al. 2017). At present, a phase II clinical trial assesses the efficacy of Exenatide in disease progression of MSA patients (NCT04431713). To summarize, the preclinical evidence supports a number of approaches for the therapy of MSA, which still await confirmation in a clinical setting.

Linked to the contribution of mutations in the COQ2 gene in rare familial MSA cases, the supplementation of coenzyme Q10 has been proposed as an individualized therapeutic approach for patients carrying the mutation/polymorphisms. A 3-year follow-up study in a MSA patient supplemented with a high-dose intake of Ubiquinol, the reduced form of coenzyme Q10, showed that the high-dose supplementation was tolerable, improved mitochondrial oxidative metabolism and the clinical rating scores remained stable over the treatment period (Mitsui et al. 2017). Currently, a phase II clinical trial with high-dose coenzyme Q10 is underway in Japanese MSA patients (R000036134).

Other preclinical studies provided rationale for MSA therapy with sodium phenylbutyrate, an unspecific histone deacetylase inhibitor tested in the PLP-α-Syn mice (Sturm et al. 2016), and benztropine, an anti-cholinergic drug acting on oligodendrocytes and enhancing re-myelination, tested in the MBP-α-Syn mice (Ettle et al. 2016). Despite the promising preclinical data, both drugs have not yet progressed to clinical trials in MSA. In the case of sodium phenylbutyrate, this has been due to expected side effects of the drug. In the case of benztropine, among other reasons, the contribution of the demyelination in early stages of the disease and its causative role for the neurodegeneration in MSA has remained uncertain. On the other hand, one of the earliest clinical trials in MSA attempted the use of recombinant human growth hormone as a “survival factor” (Holmberg et al. 2007). The treatment appeared safe and well tolerated, but no significant treatment differences for any efficacy measures were detected. Similarly, Riluzole, a benzothiazole with proposed anti-excitotoxic activity, blocking of voltage dependent sodium-channels, free-radical scavenging, anti-apoptotic and neurotrophic effects and inhibition of protein aggregation, showed borderline effect in toxin models of striatonigral degeneration and was ineffective in MSA (Diguet et al. 2005; Scherfler et al. 2005; Bensimon et al. 2009).

Cell therapies have been of interest in MSA for a long time. Initial efforts in toxin SND models have aimed at restoration of the dopaminergic response (Stefanova et al. 2005). Later on, mesenchymal stem cells (MSCs) applied intravenously were found to suppress the exacerbated neuroinflammatory cellular environment by producing anti-inflammatory cytokines and neurotrophic factors and exert neuroprotection in a transgenic MSA mouse model (Stemberger et al. 2011). The treatment with autologous MSCs has been attempted in MSA patients indicating some positive trends (Lee et al. 2008, 2012; Singer et al. 2019). However, due to some insufficiencies in the designs of these clinical trials, further studies are required and currently performed (e.g., NCT02795052, NCT02315027, NCT04876326, NCT04495582) to better evaluate the therapeutic potential of MSCs in MSA patients.

In summary, many alternative approaches to improve the cellular function and induce neuroprotection in MSA have been proposed, expanding from drug repurposing to gene and cell therapies. No reliable approach has been identified yet, despite the extensive preclinical evidence. However, many clinical trials are still underway as listed.

## Major obstacles and open questions relevant to disease modification in MSA today: concluding remarks

**Fig. 2:**
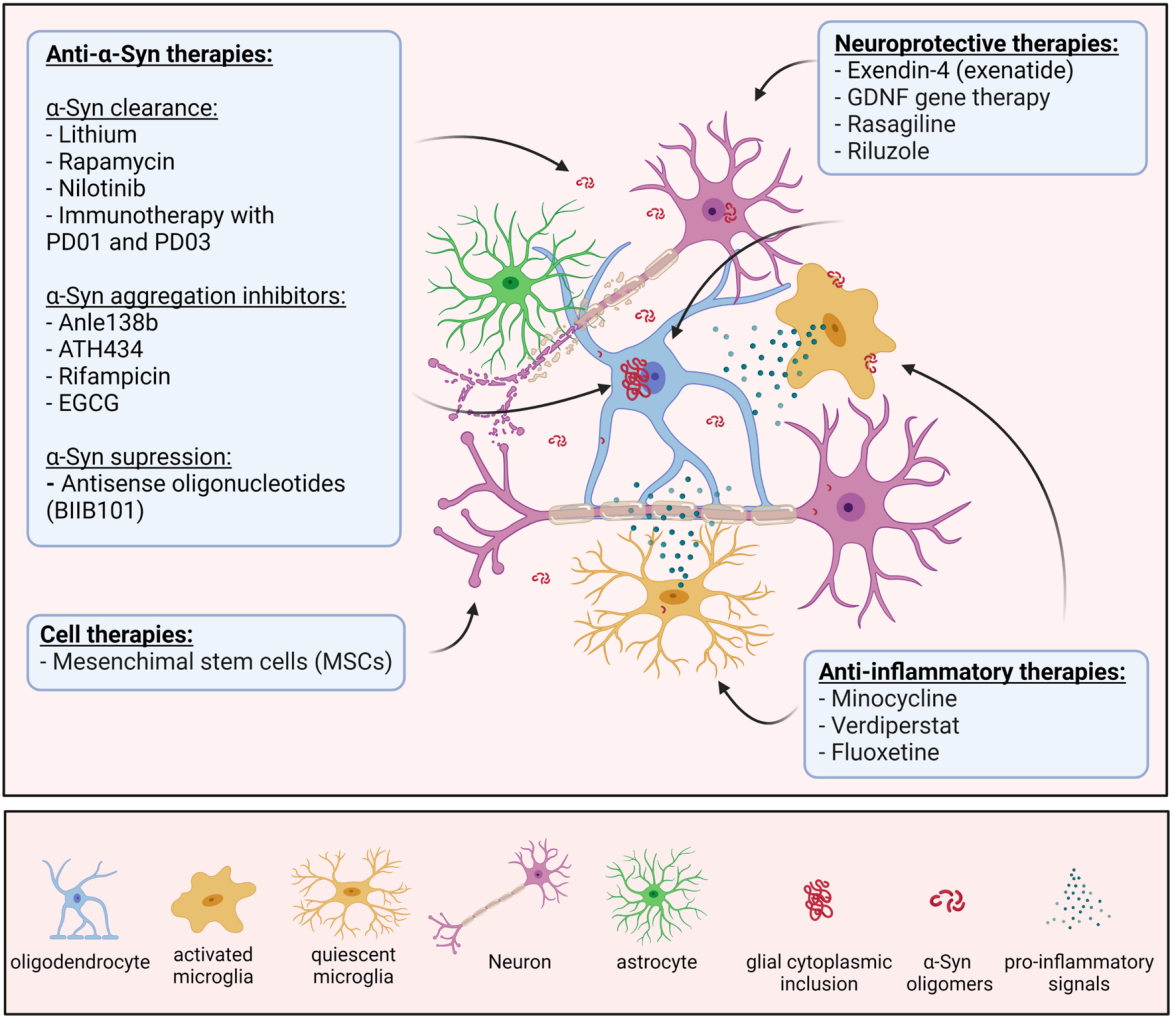
Experimental therapies in MSA clinical trials to date. Created with BioRender.com

Despite the existence of prominent therapeutic strategies (Fig. 2, Table 1) supported by extensive preclinical testing and target validation, it is important to highlight the serious gap in the translation into successful clinical trials in MSA patients. The major difficulties are linked to: (1) the challenging early diagnosis of MSA combined with the absence of useful early and progression biomarkers (Jellinger 2017); (2) the discrepancies in the design of the preclinical and clinical studies (Stefanova 2018), (3) the overambitious outcome measurements to detect changes generated by the treatment in clinical trials (Castro Caldas et al. 2017); and (4) limited knowledge about the actual trigger of MSA, and therefore, possible failure in defining the best therapeutic target(s) for disease modification.

**Table 1:** Disease-modifying therapies for MSA currently with preclinical testing and/or analysis in a clinical trial

Therapeutic approach	Therapy	Mechanisms of action	Clinical trial
Targeting α-Syn pathology	Lithium	Suggested to stimulate autophagy	A phase II clinical trial had to be discontinued due to adverse side effects (NCT0099767)
	Rifampicin	Antibiotic that showed reduced β-amyloid and α-Syn aggregation in MSA mice (Ubhi et al. 2008)	A phase III trial showed no effects of rifampicin on disease progression (NCT01287221)
	Epigallocatechin gallate (EGCG)	A poly-phenol found in green tea which reduces aggregation and toxicity of α-Syn oligomers	A phase III trial showed no effects of EGCG on disease progression (NCT02008721)
	Anle138b	Small molecule that modulates oligomerization of α-Syn in MSA mice (Heras-Garvin et al. 2019)	A phase I clinical trial with Anle138b in healthy patients was successfully completed (NCT04208152) and confirmed its safety
	ATH434 (also PBT434)	ATH434 is a moderate iron chelator that reduces α-Syn accumulation in MSA mice (Heras-Garvin et al. 2021)	A phase I clinical trial of ATH434 in healthy volunteers has been completed demonstrating the safety of the compound (ACTRN12618000541202)
	Active immunotherapy (PD01 and PD03)	Active immunotherapy targeting oligomeric α-Syn in MSA mice (Mandler et al. 2015; Lemos et al. 2020)	A phase I clinical trial suggested good immunogenicity, safety and tolerability in MSA patients (NCT02270489)
	Passive immunotherapy (rec47)	Specific antibodies are delivered to target pathological α-Syn in MSA mice (Kallab et al. 2018)	N/A
	Anti-sense oligonucleotides (ASOs)	Suppression of the production of α-Syn and therefore reduce its intracellular toxic accumulation	A phase I study to assess safety and tolerability of BIIB101, an antisense oligonucleotide targeting *SNCA*mRNA, is currently ongoing in European MSA patients (NCT04165486)
	Monophosphoryl lipid A (MPLA)	MPLA is a selective TLR4 agonist with low pro-inflammatory effect and enhancement of microglial clearance of α-Syn in MSA mice (Venezia et al. 2017)	N/A
	Molecular tweezer (CLR01)	CLR01 is a small molecule that binds to lysine residues and prevents the formation of protein assemblies in MSA mice (Herrera-Vaquero et al. 2019)	N/A
	VX-765	Caspase-1 inhibitor that inhibits α-Syn C-terminal truncation in MSA mice (Bassil et al. 2016)	N/A
	Nilotinib	c-Abl tyrosine kinase inhibitor enhancing autophagic α-Syn degradation; fails to prevent pathology in MSA mice (Lopez-Cuina et al. 2020)	Pre-clinical results did not support the development of a clinical trial
	Rapamycin	Enhances autophagic α-Syn clearance	A phase II, double-blind, futility trial was recently completed (NCT03589976)
Reducing neuroinflammation	Intravenous immunoglobulins	General anti-inflammatory effect	A single-arm interventional, single-center, open-label pilot study (NCT00750867); safe, feasible and well tolerated, no convincing efficacy
	Minocycline	A tetracycline antibiotic with brain penetrance, reduces neuroinflammation and leads to neuroprotection when therapy is initiated before motor symptom onset (Stefanova et al. 2007)	Phase III trial reduced microglial activation in MSA patients but failed to induce changes on disease progression (NCT00146809)
	Fluoxetine	Selective serotonin reuptake inhibitor that reduces neuroinflammation and leads to neuroprotection in MSA mice (Ubhi et al. 2012)	Phase II clinical trial in MSA patients did not support the progress to a phase III clinical trial (NCT01146548)
	Verdiperstat (BHV3241, previously AZD3241)	Oral myeloperoxidase (MPO) inhibitor that reduces neuroinflammation; shows neuroprotection only after early start of therapy in MSA mice, not efficient when started after motor symptom onset (Stefanova et al. 2012; Kaindlstorfer et al. 2015)	Phase III clinical trial recently completed (NCT03952806), no results published yet
Neuroprotective therapies	Recombinant human growth hormone	General trophic effects	A double-blind, placebo-controlled study (Holmberg et al. 2007) was underpowered and failed to show change in efficacy measurements
	Riluzole	Benzothiazole with proposed anti-excitotoxic activity, blocking of voltage dependent sodium-channels, free-radical scavenging, anti-apoptotic and neurotrophic effects and inhibition of protein aggregation, borderline effect in toxin models of striatonigral degeneration (Scherfler et al. 2005; Diguet et al. 2005)	A Phase III study on MSA patients showed no effect on disease progression (NCT00211224)
	Rasagiline	Irreversible inhibitor of monoamine oxidase-B with anti-apoptotic effect through proposed neurotrophic support, neuroprotection in MSA mice (Stefanova et al. 2008)	A phase II trial in MSA patients did not show significant benefits when used in a clinically applicable dose (NCT00977665)
	Exenatide (Exendin-4)	Anti-diabetic drug and a glucagon-like peptide-1 analogue targeting brain insulin resistance showing protection of dopaminergic neurons but no motor improvement in MSA mice (Bassil et al. 2017)	Phase II clinical trial recently completed (NCT04431713), no results published yet
	Sodium phenylbutyrate	Unspecific histone deacetylase, shows neuroprotection and lowering of α-Syn through modified epigenetic control in MSA mice (Sturm et al. 2016)	N/A
	Benztropine	Anti-cholinergic drug acting on oligodendrocytes and enhancing re-myelination, efficient in MSA mice (Ettle et al. 2016)	N/A
	AAV2-GDNF	Neurotrophic support leading to neuroprotection in MSA mice (Ubhi et al. 2010)	Phase I clinical trial on GDNF gene therapy in progress (NCT04680065)

*N/A* not available

In summary, the last two decades have laid the basis of the active search for MSA disease-modifying therapies. Significant knowledge has been generated on understanding the disease mechanisms, which made it possible to identify α-Syn pathology and glial responses as significant targets to modulate the progression of the neurodegenerative process. Facing the above-mentioned challenges, we enter a new stage of fine-tuning and adaptation of successful experimental therapies to more suitable trial designs, which will lead to stopping or slowing the progression of MSA. New MSA preclinical models may be an important step towards refined drug screening and improved translation between preclinical and clinical studies. Since MSA is a multifactorial disease, the use of combined multi-target individualized therapies might constitute an important advantage in future design of clinical trials on disease modification.

## Data Availability

Not applicable.
